# Sex-specific associations between Mediterranean Diet Score and long-term risk of mortality, dementia and other chronic diseases. 20-year evidence from a Swedish population study of 70-year old individuals

**DOI:** 10.1016/j.jnha.2026.101012

**Published:** 2026-10-28

**Authors:** Gianluca Tognon, Valter Sundh, Elisabet Rothenberg, Jessica Samuelsson, Silke Kern, Ingmar Skoog, Lauren Lissner

**Affiliations:** a School of Health Sciences, University of Skövde, Skövde, Sweden; b School of Public Health and Community Medicine, Institute of Medicine, Sahlgrenska Academy at the University of Gothenburg, Sweden; c Department of Nursing and Integrated Health Sciences, Kristianstad University, Kristianstad, Sweden; d Neuropsychiatric Epidemiology Unit, Department of Psychiatry and Neurochemistry, Institute of Neuroscience and Physiology, Sahlgrenska Academy at the University of Gothenburg, Sweden

**Keywords:** All-Cause mortality, Cardiovascular disease, Dementia, Mediterranean Diet Score, Older adults

## Abstract

**Objectives:**

To investigate the association between adherence to the Mediterranean Diet Score (MDS) and the risk of all-cause mortality, cardiovascular disease (CVD), cancer, and dementia morbidity over 10- and 20-year follow-up periods in a population-based cohort of older adults.

**Design:**

A prospective population-based cohort study.

**Setting:**

Gothenburg, Sweden.

**Participants:**

A total of 1298 participants (56.2% women) aged 70 years at baseline from the general population and recruited from the Gothenburg H70 and Population Study of Women in Gothenburg (PSWG) birth cohorts.

**Measurements:**

Dietary intake was assessed via face-to-face, semi-structured diet history interviews. The MDS was calculated based on eight food groups (excluding alcohol). Outcomes were identified through linkage with national health and death registers. Cox proportional hazard models were used, adjusting for BMI, smoking, marital status, physical activity, and education.

**Results:**

A higher MDS was associated with a lower risk of 20-year dementia morbidity in men (multivariable-adjusted HR = 0.83; 95% CI: 0.71–0.96), but not in women. This association did not remain statistically significant after correction for multiple testing, and the sex-by-MDS interaction (p = 0.048) was nominally significant, but not robust to multiple testing. No statistically significant associations were found for CVD or cancer morbidity in either sex over the available follow-up (i.e., 10 and 20 years, and up to 18 years for cancer; 20-year CVD follow-up in men: HR = 0.98; 95% CI: 0.88–1.08). For all-cause mortality, an initial association in men at 10 years (HR = 0.90; 95% CI: 0.82–0.99) was no longer significant after full adjustment (HR = 0.92; 95% CI: 0.84–1.01).

**Conclusions:**

Higher MDS levels were associated with lower dementia risk in men, but this finding was not robust to multiple testing correction and requires confirmation in larger studies. No association was found with all-cause mortality, CVD and cancer morbidity. A limitation of these findings is that dietary assessment was conducted at a single time point (age 70), and that dementia ascertainment relied on registers which are estimated to miss > 50% of cases, particularly among home-dwellers.

## 1 Introduction

The global burden of age-related chronic non-communicable diseases, including cardiovascular diseases (CVD), various cancers, and neurodegenerative disorders such as dementia, represents a significant public health challenge, with an immense and projected increase in incidence with population aging [1]. In the absence of definitive pharmacological treatments, there is a compelling rationale for focusing on modifiable risk factors, with diet playing a pivotal role in influencing key health indicators such as blood pressure, lipid profiles, obesity, inflammation, and endothelial function [2]. Unhealthy dietary patterns are clearly implicated in the development and progression of these conditions [3].

The Mediterranean diet (MD) is a widely recognized healthy eating pattern traditionally observed in the olive-growing regions of the Mediterranean basin, now globally endorsed by leading health organisations [4]. It is a plant-based pattern rich in vegetables, fruits, legumes, seeds and nuts, and includes a moderate consumption of fish, poultry, and wine (typically with meals), and a limited intake of red and processed meats, dairy products, and sweets [5].

This dietary pattern is recognized for its anti-inflammatory and antioxidant properties, attributed to its rich content of fiber, phytochemicals, polyphenols, and healthy fats [6]. Accumulating evidence consistently demonstrates the MD's protective effects against a wide range of chronic diseases, leading to reduced overall mortality and longer survival [7]. Specifically, high adherence to the MD has been associated with a reduction in all-cause mortality [8], a lower risk of cardiovascular disease (CVD) incidence and mortality [9], including stroke and heart failure [10], and a reduced risk of overall cancer incidence and specific cancer types, such as breast, colorectal, gastric, and lung cancers [11], a lower incidence of dementia, mild cognitive impairment, and Alzheimer's disease (AD), alongside slower cognitive decline [12].

Adherence to the MD is frequently assessed using validated scoring tools, calculated based on the consumption frequency of characteristic food groups. Example of such scores include the Mediterranean Diet Score (MDS), which we used previously in cohort studies of adults [13] and children [14], and the more recent Mediterranean Diet Adherence Screener (MEDAS) [15].

Although adherence to the Mediterranean diet has been associated with lower risks of mortality, cardiovascular disease, cancer, and dementia, important uncertainties remain regarding its relevance in advanced old age. Recent meta-analyses in adults who are 60 years or older confirmed lower all-cause and cardiovascular mortality but highlighted substantial heterogeneity in study populations, dietary assessment methods, and follow-up durations [16]. Existing evidence is still dominated by cohorts enrolling younger-old adults, with relatively few studies including substantial numbers of participants aged 80 years or older or examining outcomes over follow-up periods extending beyond 10 years. Consequently, it remains unclear whether adherence to the Mediterranean diet continues to confer protection into very late life, when competing morbidity, frailty, and age-related physiological changes may modify diet-disease associations. Furthermore, most prior studies have focused on single endpoints, limiting the ability to evaluate whether associations with mortality, dementia, and chronic disease are consistent within the same population and methodological framework. Evidence for dementia remains particularly inconsistent, with both inverse and null associations reported across cohort studies and meta-analyses [17]. One important limitation of previous studies is that relatively short follow-up periods may increase susceptibility to reverse causality, as dietary habits can change during the prodromal phase of cognitive decline [18]. By leveraging up to 20 years of follow-up across multiple H70 birth cohorts, the present study examines long-term associations between Mediterranean diet adherence and mortality, dementia, and chronic disease risk in adults aged 70 years and older. This extended follow-up may attenuate, though not fully remove, the influence of preclinical disease-related changes in diet.

Potential sex differences in associations between dietary patterns and age-related health outcomes remain insufficiently understood. Biological differences in metabolism, hormonal regulation, vascular risk, and immune function may influence how men and women respond to dietary exposures across aging [19]. Some studies have reported stronger protective associations between Mediterranean diet adherence and cardiovascular disease or mortality in women, whereas findings for dementia and cognitive outcomes remain inconsistent [12]. However, evidence in very old adults is scarce. Investigating potential sex-specific associations may therefore improve understanding of dietary influences on healthy aging and help inform targeted prevention strategies.

Although we had no specific hypothesis on the direction of any interaction, sex-stratified analyses were planned a priori because sex differences are well documented both in dementia incidence and in dietary behaviour in this age group [20]. Observational studies have frequently described a J-shaped association between alcohol intake and several health outcomes, and moderate consumption is a traditional component of the Mediterranean diet. However, much of the apparent benefit reflects confounding and abstainer misclassification as evidenced by the Nordic Nutrition Recommendations 2023 scoping review [21]. Therefore, alcohol intakes were not included in MDS calculations. This study builds on a previous paper that reported an inverse association between adherence to a Mediterranean-like diet and all-cause mortality at 10 years in the H70 and PSWG prospective cohorts of 70-year-old Swedes living in Gothenburg, Sweden [22]. The primary aim, defined a priori, was to examine sex-specific associations between the MDS and the long-term risk of all-cause mortality and of cardiovascular, cancer and dementia morbidity, comparing men and women. Analyses involving APOE genotype were planned a priori as secondary, and analyses using the alcohol-inclusive MDS were pre-defined but are considered exploratory, as they required exclusion of one birth cohort.

## 2 Subjects and methods

### 2.1 Subjects and dietary assessment

This study is based on the Gothenburg H70 prospective birth cohort study [[23], [24], [25], [26]] and the Population Study of Women in Gothenburg (PSWG). Similar methodologies were used in all waves of the two surveys. However, over the years new questions were at times added while keeping the original questions unchanged with few exceptions. The latter included secular changes in the diet and other lifestyle factors, where some items were replaced by more recent ones or new items were added. In such cases minor adjustments were made to harmonize answers in all cohorts. The study participants were 70 years old in 1971, 1981, 1992 or 2000, at which time they completed questionnaires and underwent physical examinations and dietary interviews.

Diet history assessments were completed through face-to-face, semi-structured interviews conducted by a trained dietician. Interviews typically lasted about 60–90 min and included detailed questions on usual food frequency and portion sizes, aided by visual portion-size models to capture habitual dietary intake over the preceding three months [23]. Dietary data was validated by comparing energy intake with total energy expenditure, measured objectively using heart rate monitors, diaries, and doubly-labelled water [26].

The base sample consisted of 1381 persons with dietary interviews at age 70. To minimize reporting bias, 1298 individuals were included in the analyses after excluding the 5% most extreme energy intake values in relation to basal metabolic rate (EI/BMR) ratio (which corresponded to the following cut-offs: < 0.891 and > 2.223) or missing values for BMI. BMR was estimated via standard equations (males: 0.0499 × weight (kg) + 2.93; females: 0.0386 × weight (kg) + 2.875; [27]). The sample of 1298 participants (730 women, 56.2%) constituted the analytic sample described in Table 1a, Table 1b and was used for the birth-cohort-adjusted (basic) models. Fully adjusted models additionally required complete information on smoking (never smoker or having stopped more than 10 years ago), education, physical activity, and marital status; 69 participants (5.3%) had missing data on one or more of these covariates, leaving 1229 participants (696 women, 533 men) for the fully-adjusted analyses. Missing data on individual covariates were as follows: education 4.0% (n = 52), physical activity 1.2% (n = 15), marital status 0.3% (n = 4), and smoking 0.2% (n = 3); body mass index was complete, as participants with missing BMI had already been excluded. Education missingness was concentrated in the 1922 birth cohort (23.6%). Because the same covariate set was used for all outcomes, the 69 participants (5.3%) with missing covariate data were excluded from every fully adjusted model; these exclusions were therefore identical across outcomes and not outcome-dependent. The primary analyses were based on complete cases; multiple imputation was used as a sensitivity analysis (see below).

**Table 1a:** Descriptive of all-cause mortality, CVD, cancer, and dementia cases, at 10 years follow-up stratified by birth cohort and sex.

	% participants (n subjects)				
	Birth cohorts				Total
	1901−2	1911−2	1922	1930	
*Females*					
Deceased	20.4 (n = 35)	23.2 (n = 29)	19.4 (n = 24)	12.6 (n = 39)	17.4 (n = 127)
CVD cases^a^	13.4 (n = 23)	30.4 (n = 38)	21.0 (n = 26)	20.7 (n = 64)	20.7 (n = 151)
Cancer cases^a^	18.6 (n = 32)	21.6 (n = 27)	30.6 (n = 38)	31.1 (n = 96)	26.4 (n = 193)
Dementia cases^a^	1.7 (n = 3)	4.8 (n = 6)	4.0 (n = 5)	5.5 (n = 17)	4.2 (n = 31)
**Participants recruited**	**100 (n = 172)**	**100 (n = 125)**	**100 (n = 124)**	**100 (n = 309)**	**100 (n = 730)**
*Males*					
Deceased individuals	50.6 (n = 89)	48.0 (n = 59)	25.9 (n = 14)	23.3 (n = 50)	37.3 (n = 212)
CVD cases^a^	19.9 (n = 35)	30.9 (n = 38)	20.4 (n = 11)	23.3 (n = 50)	23.6 (n = 134)
Cancer cases^a^	24.4 (n = 43)	23.6 (n = 29)	35.2 (n = 19)	37.7 (n = 81)	30.3 (n = 172)
Dementia cases^a^	2.3 (n = 4)	3.3 (n = 4)	1.9 (n = 1)	4.7 (n = 10)	3.3 (n = 19)
Participants recruited	100 (n = 176)	100 (n = 123)	100 (n = 54)	100 (n = 215)	100 (n = 568)

a Register-based incident cases.

**Table 1b:** Descriptive of all-cause mortality, CVD, cancer, and dementia cases, at 20 years follow-up stratified by birth cohort and sex.

	% participants (n subjects)				
	Birth cohorts				Total
	1901−2	1911−2	1922	1930	
*Females*					
Deceased individuals	75.6 (n = 131)	68.8 (n = 86)	61.3 (n = 76)	55.3 (n = 171)	63.4 (n = 463)
CVD cases^a^	43.0 (n = 74)	59.2 (n = 74)	46.0 (n = 57)	31.1 (n = 96)	41.2 (n = 301)
Cancer cases^a^	27.9 (n = 48)	31.2 (n = 39)	36.3 (n = 45)	43.7 (n = 135)	36.6 (n = 267)
Dementia cases^a^	14.0 (n = 24)	27.2 (n = 34)	16.1 (n = 20)	15.5 (n = 48)	17.3 (n = 126)
**Participants recruited**	**100 (n = 172)**	**100 (n = 125)**	**100 (n = 124)**	**100 (n = 309)**	**100 (n = 730)**
*Males*					
Deceased individuals	91.5 (n = 161)	92.7 (n = 114)	70.4 (n = 38)	71.6 (n = 154)	82.2 (n = 467)
CVD cases^a^	36.9 (n = 65)	48.8 (n = 60)	38.9 (n = 21)	29.3 (n = 63)	36.8 (n = 209)
Cancer cases^a^	30.7 (n = 54)	31.7 (n = 39)	44.4 (n = 24)	48.8 (n = 105)	39.1 (n = 222)
Dementia cases^a^	6.8 (n = 12)	19.5 (n = 24)	18.5 (n = 10)	12.6 (n = 27)	12.9 (n = 73)
**Participants recruited**	**100 (n = 176)**	**100 (n = 123)**	**100 (n = 54)**	**100 (n = 215)**	**100 (n = 568)**

a Register-based incident cases.

To verify that exclusion of energy-intake outliers did not introduce selection bias, excluded (n = 71) and included (n = 1298) participants were compared on baseline characteristics (i.e., sex, birth cohort, education, marital status, smoking, physical activity, body mass index and the two versions of the Mediterranean Diet Score) using chi-square tests for categorical variables and permutation t-tests for continuous variables. In particular, in the 1922 cohort, missing education data were initially collected via a follow-up telephone interview (54.5% response) and supplemented by a re-examination conducted in 2000, raising the response rate to 76.4%. The remaining 42 participants (23.6% of the 1922 cohort) with missing education data (together with 10 participants with missing education in the other birth cohorts, 52 in total) were excluded from models including education as a covariate.

Sample sizes varied for specific outcomes; for instance, participants with a cancer diagnosis or hospital admission for cardiovascular disease before the baseline examination were excluded from those respective incidence analyses. Detailed descriptions of the initial recruitment methods for the health examinations are provided in earlier publications, such as [25,26].

### 2.2 The Mediterranean Diet Score (MDS)

The MDS was operationalised by mapping the conventional MDS components to the food variables available from the diet history interviews. For each MDS component, all relevant dietary interview variables were combined into a predefined food group, energy-adjusted, and scored according to sex-specific medians. Energy adjustment was performed using intakes expressed per 1000 kcal, applied in the same way to all food groups. The food groups used for the score calculations were vegetables and potatoes, fruit and fresh fruit juices, legumes, nuts, and seeds, wholegrain cereals, fish and shellfish, the ratio between total unsaturated and saturated fats, meat, processed meat, and eggs, and dairy products. Intakes of the latter were calculated from all available dairy variables, including milk, soured milk, yoghurt, cream, cheese, and cottage cheese/quark, irrespective of fat content. For all food groups except meat and dairy products, one point was assigned for energy-adjusted intakes higher than sex-specific medians, whereas the opposite was done for meat and dairy products. Supplementary Table 1 reports a detailed description of how each food group was constructed from the available dietary variables as well as intakes of the food groups and the fat ratio included in the MDS calculations. The latter included food groups scored positively such as: 1. whole grain cereals, fruit and fresh juices, 2. vegetables and potatoes, 3. fish and seafood, 4. legumes, nuts and seeds, and 5. a ratio between unsaturated (both mono- and polyunsaturated) and saturated fat and items scored negatively, such as 1. dairy products and 2. meat, meat products and eggs. Potatoes were considered a staple vegetable component of the Swedish diet. An alternative score, considering moderate intakes of alcoholic beverages, was also calculated as has frequently been done in earlier research [22,28,29]. This version assigned 1 point to intakes above the population median, which in this cohort corresponded to moderate alcohol consumption and is consistent with the scoring approach used in other epidemiological studies of Mediterranean diet adherence.

### 2.3 APOE genotyping

Considering its role in determining the future risk of dementia, an exploratory analysis based on APOE genotyping was also conducted. Odds ratios for dementia by APOE-ε4 status within MDS groups were estimated using unadjusted logistic regression, given the small number of dementia cases in these subgroups. Dementia cases were identified from the Swedish National Patient Register during the 20-year follow-up.

Genotyping for Apolipoprotein E (APOE) was performed using DNA extracted from blood samples collected at the baseline examination. The APOE-ε4 polymorphism was determined using standardised polymerase chain reaction (PCR) techniques to identify the ε2, ε3, and ε4 alleles. For the purpose of this analysis, participants were categorized as APOE-ε4 carriers if they possessed at least one ε4 allele (heterozygous or homozygous). This information was available only in a sub-sample of 600 participants who had both dietary and genetic data available. Of these, 213 were men from the 1930 (examined in 2000) birth cohort and 387 were women from two distinct cohorts i.e., 86 women from the 1922 birth cohort (examined in 1992) and 301 women from the 1930 birth cohort (examined in 2000).

### 2.4 Endpoints

Data from the examinations was linked to national registers based on participants’ Swedish personal number. All-cause mortality data were obtained from the national cause of death register, whereas CVD (first cardiovascular event only) and dementia incidence outcomes were identified using both the in-patient hospital register and the death register. Cancer incidence data were sourced from the cancer register, with available follow-up limited to 18 years.

### 2.5 Statistical analyses

Follow-up was censored at the date of death or at the end of the 10 and 20-year follow-up period. Due to the high-quality linkage with the Swedish National Health and Cause of Death Registers, loss to follow-up was negligible.

Cox proportional hazard models using all-cause mortality and incidence of cardiovascular, cancer, and dementia morbidity as outcomes were used. Time was modelled as days from examination to event or to censoring (in case of death) and the proportional hazard assumption was assessed for all models using Schoenfeld residuals, with p < 0.05 taken as evidence of a potential violation. Two models were compared, one only adjusted by birth cohort and one multivariable-adjusted model (also including BMI calculated from measured body weight and height, and the following self-reported variables: smoking status, physical activity, marital status, education, and smoking history i.e., never smoker or stopped > 10 years ago). Given the age of the participants and the historical period covered by the cohort, marital status was considered a reasonable proxy for living situation. Many participants from the 1922 cohort lacked information on education (23.6%), though available data for this group was obtained from follow-up phone calls. We analysed each endpoint at both 10 and 20 years follow-up. Analyses were stratified by sex.

Hazard ratios from the fully adjusted models for all outcomes, both sexes and both follow-up periods were summarised in a forest plot.

The cumulative incidence of dementia over 20 years, calculated as one minus the Kaplan-Meier survival estimate, was displayed by MDS tertile separately for women and men.

Correction for multiple testing was applied using the Benjamini-Hochberg false discovery rate across the family of 16 fully adjusted models (4 outcomes x 2 follow-up periods x 2 sexes), using the p-value for the MDS term from each model. The birth-cohort-adjusted (basic) models and the sex-by-MDS interaction tests were not included in this family; they are reported with unadjusted p-values and interpreted descriptively. Including them in the correction family would not change any conclusion, as no association would remain statistically significant.

We assessed statistical precision by estimating the minimum detectable effect. This approach is considered more appropriate than traditional post-hoc power analyses, which are largely determined by the observed result [30]. For the association between higher MDS adherence and dementia in men, the estimate with the fewest events (n = 70) and therefore the most limited precision, we computed the smallest hazard ratio, per one-point increment in the MDS, detectable with 80% power at a two-sided α of 0.05. This followed Hsieh and Lavori's approach [31] for a continuous covariate in Cox regression, accounting for the standard deviation of the MDS and the proportion of its variance explained by the remaining covariates.

Sensitivity analyses were conducted in men to test the association between the risk of dementia and an alternative version of the Mediterranean Diet Score in which alcohol intakes were also considered. In these analyses, the oldest cohort (1901−2) was excluded, as alcohol-related survey questions differed significantly from those used in subsequent recruitments. The association between MDS, APOE-ε4 status, and the risk of dementia was also analysed. However, due to the limited number of subjects for which this information was available, we consider this analysis as purely exploratory in view of future analyses on a larger dataset.

Sex-by-diet interaction terms (MDS x sex) were added to combined-sample (men and women) fully adjusted Cox models for each outcome and follow-up period to formally test for effect modification by sex. Interaction p-values below 0.05 were considered indicative of significant effect modification.

The sex-stratified models for dementia incidence were additionally re-estimated with further adjustment for hypertension (drug treatment for hypertension, diastolic blood pressure) and diabetes (diagnosed diabetes, fasting/random blood glucose). Participants with missing data on one or more of these covariates were excluded from this analysis (n = 15 in each sex), yielding analytic samples of 553 men (69 dementia events) and 715 women (122 dementia events).

To test whether the association between MDS and dementia remained constant over the follow-up period, we refitted the Cox models including a time-varying coefficient for the MDS, that is, an interaction between the MDS and follow-up time. To assess the influence of missing covariate data (69 participants, 5.3%; education missing for 4.0% of participants overall and for 23.6% of the 1922 birth cohort), we performed multiple imputation of all covariates with missing values as a sensitivity analysis. Imputation was performed separately in men and women and for each outcome, by predictive mean matching (20 imputations), with an imputation model including all covariates, the MDS, the event indicator and the Nelson-Aalen estimate of the cumulative hazard [32]; estimates from the fully adjusted models were combined across imputations using Rubin's rules. To address possible reverse causality arising from dietary changes during the prodromal phase of dementia, we performed a landmark sensitivity analysis in which participants recorded in registers with dementia during the first five years of follow-up were excluded, and the association with the MDS was re-estimated among those still free of dementia at that point.

As a final sensitivity analysis, the fully adjusted models were repeated in the full sample retaining the participants excluded for implausible energy reporting (n = 1369).

### 2.6 Ethics

The examinations of 70-year-olds in the H70 study and the Population Study of Women in Gothenburg from 1992 and 2000 have been approved by the Gothenburg University Ethics Committee in accordance with the Declaration of Helsinki (1989) of the World Medical Association. All participants were informed of the aims and procedures of the study and gave their consent.

## 3 Results

### 3.1 Descriptive analyses

The number and proportion of participants recruited in each birth cohort and the number of deaths, CVD, cancer, and dementia cases are reported in Tables 1a (10-year follow-up) and 1b (20-year follow-up), stratified by birth cohort and sex. Mortality was consistently higher among males than females across all cohorts and increased from 17.4% to 63.4% in women and from 37.3% to 82.2% in men in the longer follow-up. This data also shows longer survival rates from the earliest-born cohort to the most recent ones. At 10 years follow-up, the proportion of CVD cases was higher in males than females for the oldest and youngest cohort but similar in the other two cohorts. At 20 years, women showed a higher CVD incidence in all cohorts, likely reflecting longer survival compared to men. The 1930 birth cohort contributed with both the largest number of participants and proportion of CVD and cancer cases. Cancer incidence was higher at 20 years than at 10 years, and higher among males compared to females.

Dementia incidence showed a distinct birth cohort effect; at 20 years of follow-up, the cumulative incidence was substantially lower in the most recent cohort (1930) compared to earlier ones (e.g., 1911−12), falling from 27.2% to 15.5% in women and from 19.5% to 12.6% in men. While incidence increased across all groups between the 10 and 20-year follow-up, the rise was more pronounced in the earliest-born cohorts. Overall, females maintained a higher cumulative incidence of dementia compared to men across most cohorts (17.3% vs 12.9% at 20 years).

Tables 2a (females) and 2b (males) provide the means and standard deviations as well as the median and interquartile range (IQR) of the Mediterranean diet score, calculated excluding alcohol intakes, across birth cohorts. Adherence to the Mediterranean diet was similar among men and women (median [IQR] = 4 [3–5] in men, women and when calculated based on data from all participants), with comparable variability, but it increased in more recently examined cohorts. Descriptive statistics for key confounders including physical activity, marital status, education level, smoking status, and BMI classification are also reported. Descriptively, later-born cohorts appeared to have higher levels of physical activity and education in most cohorts, and lower smoking rates. Males were more often classified as married, whereas females were more often classified as widows. Females also appeared more likely than men to maintain a normal body weight.

**Table 2a:** Descriptive of the Mediterranean diet score (MDS), calculated without alcohol intakes (exposure) and the covariates included in the Cox models as potential confounding factors, stratified by birth cohort in women.

Covariates	1901−2	1911−2	1922	1930	All
MDS, mean (SD)	3.4 ± 1.4	3.3 ± 1.4	4.2 ± 1.4	4.5 ± 1.6	**4.0 ± 1.6**
MDS, median (Q1–Q3)	3 (2−4)	3 (2−4)	4 (3−5)	5 (3−6)	**4 (3–5)**
Physically active, n (%)	80.8 (n = 139)	68.8 (n = 86)	79.8 (n = 99)	90.9 (n = 281)	**82.9 (n = 605)**
Married, n (%)	47.7 (n = 82)	43.2 (n = 54)	55.6 (n = 69)	49.5 (n = 153)	**49.0 (n = 358)**
Education above basics, n (%)	15.7 (n = 27)	20.8 (n = 26)	31.5 (n = 39)	37.2 (n = 115)	**28.4 (n = 207)**
Never smoker (or stopped > 10 years ago), n (%)	84.3 (n = 145)	84.8 (n = 106)	68.5 (n = 85)	74.8 (n = 231)	**77.7 (n = 567)**
Underweight, n (%)	1.2 (n = 2)	1.6 (n = 2)	1.6 (n = 2)	0.3 (n = 1)	**1.0 (n = 7)**
Normal weight, n (%)	45.3 (n = 78)	33.6 (n = 42)	46.0 (n = 57)	33.7 (n = 104)	**38.5 (n = 281)**
Overweight, n (%)	37.2 (n = 64)	42.4 (n = 53)	33.9 (n = 42)	42.1 (n = 130)	**39.6 (n = 289)**
Obese, n (%)	16.3 (n = 28)	22.4 (n = 28)	18.5 (n = 23)	23.9 (n = 74)	**21.0 (n = 153)**
**All females, n (%)**	**100 (n = 172)**	**100 (n = 125)**	**100 (n = 124)**	**100 (n = 309)**	**100 (n = 730)**

**Table 2b:** Descriptive of the Mediterranean diet score (MDS), calculated without alcohol intakes (exposure) and the covariates included in the Cox models as potential confounding factors, stratified by birth cohort in men.

Covariates	1901−2	1911−2	1922	1930	All
MDS, mean (SD)	3.8 ± 1.4	3.6 ± 1.5	4.2 ± 1.6	4.3 ± 1.6	**4.0 ± 1.5**
MDS, median (Q1–Q3)	4 (3−5)	4 (3−4)	4 (3−6)	4 (3−5)	**4 (3–5)**
Physically active, n (%)	89.8 (n = 158)	82.9 (n = 102)	88.9 (n = 48)	88.4 (n = 190)	**87.7 (n = 498)**
Married, n (%)	78.4 (n = 138)	78.0 (n = 96)	72.2 (n = 39)	74.0 (n = 159)	**76.1 (n = 432)**
Education above basics, n (%)	17.6 (n = 31)	26.0 (n = 32)	29.6 (n = 16)	42.8 (n = 92)	**30.1 (n = 171)**
Never smoker (or stopped > 10 years ago), n (%)	40.9 (n = 72)	52.8 (n = 65)	66.7 (n = 36)	74.9 (n = 161)	**58.8 (n = 334)**
Underweight, n (%)	1.7 (n = 3)	1.6 (n = 2)	0 (n = 0)	0.5 (n = 1)	**1.1 (n = 6)**
Normal weight, n (%)	46.0 (n = 81)	36.6 (n = 45)	27.8 (n = 15)	31.6 (n = 68)	**36.8 (n = 209)**
Overweight, n (%)	43.8 (n = 77)	51.2 (n = 63)	53.7 (n = 29)	48.4 (n = 104)	**48.1 (n = 273)**
Obese, n (%)	8.5 (n = 15)	10.6 (n = 13)	18.5 (n = 10)	19.5 (n = 42)	**14.1 (n = 80)**
**All males, n (%)**	**100 (n = 176)**	**100 (n = 123)**	**100 (n = 54)**	**100 (n = 215)**	**100 (n = 568)**

Excluded and included participants were comparable on sex, birth cohort, education, marital status, smoking, mean body mass index and, importantly, the Mediterranean Diet Score itself (all p ≥ 0.05; Supplementary Table 2). The only difference was a modestly higher proportion of low physical activity among excluded participants (25.4% vs 13.9%, p = 0.014), consistent with the known association between implausible energy reporting and lower physical activity. As the exposure did not differ between groups, this exclusion is unlikely to have biased the diet–outcome associations.

### 3.2 All-cause mortality

Table 3 shows HRs and CIs from Cox proportional hazard models, assessing the association between the MDS and all-cause mortality, stratified by sex. Both unadjusted and adjusted p-values for multiple testing are also included. In women, the score calculated without including alcohol intakes did not show any statistically-significant association at either 10 or 20 years. However, in men a statistically-significant association was present at 10 years follow-up in the model only adjusted by birth cohort (HR = 0.903, 95% CI: 0.825−0.988), but not in the multivariable-adjusted models. No statistically-significant associations were found after 20 years. Also, no p-values for the mortality analyses remained significant after adjusting for multiple testing (Benjamini-Hochberg false discovery rate).

**Table 3:** Cox-proportional hazard models, assessing the association between the Mediterranean Diet Score (MDS) and the risk of all-cause mortality at 10 and 20 years of follow-up, either adjusted for birth cohort only or for potential confounders and stratified by sex. Hazard Ratios (HR) represent the risk associated with a one-unit increase in the MDS. p-values are presented both unadjusted and adjusted for multiple testing (false discovery rate).

	HRs (95% CIs)									
*10 years*						*20 years*				
*Women*	n (events)	HR	CIs	p-Value (unadjusted)	p-Value (adjusted)	n (events)	HR	CIs	p-Value (unadjusted)	p-Value (adjusted)
Adjusted by birth cohort only	730 (127)	0.971	0.866 – 1.089	0.618	–	730 (464)	0.970	0.915 – 1.028	0.306	–
Multivariable-adjusted^a^	696 (113)	0.995	0.878 – 1.127	0.936	0.936	696 (440)	0.975	0.917 – 1.036	0.413	0.826
*Men*										
Adjusted by birth cohort only	568 (212)	0.903	0.825 – 0.988	0.026	–	568 (467)	0.955	0.899 – 1.014	0.131	–
Multivariable-adjusted^a^	533 (202)	0.919	0.836 – 1.009	0.078	0.619	533 (440)	0.959	0.901 – 1.021	0.194	0.826
*p for interaction sex x MDS*		0.307					0.577			

a Also adjusted for BMI, smoking history (never smoker or stopped > 10 years ago), marital status, physical activity, education.

### 3.3 Chronic disease incidence

Table 4 shows the results of the analyses of the association between the MDS and, respectively, CVD, cancer, and dementia incidence. Both unadjusted and adjusted p-values for multiple testing are also included. All analyses on the association between MDS and CVD morbidity (first cardiovascular event) were statistically non-significant (e.g., women, 20 year follow-up: HR 0.963, 95% CI 0.891–1.041, men: HR = 0.975, 95% CI 0.882–1.077). Minimum changes in HRs between a basic model (adjusted for year of birth, and sex when men and women were combined) and a full model (including all covariates used in previous analyses) were observed. The results were consistent between men and women, although the HRs were slightly lower at 10 years.

**Table 4:** Cox-proportional hazard models, assessing the association between the Mediterranean Diet Score (MDS) and the risk of CVD, cancer, and dementia incidence at 10 and 20 years of follow-up, either adjusted for birth cohort only or for potential confounders and stratified by sex. Hazard Ratios (HR) represent the risk associated with a one-unit increase in the MDS. p-values are presented both unadjusted and adjusted for multiple testing (false discovery rate).

	HRs (95% CIs)									
	*10 years*					*20 years*				
CVD incidence	n (events)	HR	CIs	p-Value (unadjusted)	p-Value (adjusted)	n (events)	HR	CIs	p-Value (unadjusted)	p-Value (adjusted)
*Women*										
Adjusted by birth cohort only	730 (151)	0.931	0.841 – 1.030	0.166	–	730 (301)	0.961	0.893 – 1.035	0.292	–
Multivariable-adjusted^a^	696 (144)	0.946	0.850 – 1.052	0.304	0.826	696 (286)	0.963	0.891 – 1.041	0.344	0.826
*Men*										
Adjusted by birth cohort only	568 (134)	0.935	0.832 – 1.051	0.261	–	568 (209)	0.969	0.882 – 1.064	0.507	–
Multivariable-adjusted^a^	533 (125)	0.945	0.834 – 1.071	0.375	0.826	533 (195)	0.975	0.882 – 1.077	0.619	0.879
*p for interaction sex x MDS*		0.727					0.643			
**Cancer incidence**										
*Women*										
Adjusted by birth cohort only	730 (193)	0.997	0.911 – 1.091	0.947	–	730 (267)	0.996	0.923 – 1.075	0.918	–
Multivariable-adjusted^a^	696 (183)	0.989	0.901 – 1.087	0.824	0.879	696 (256)	0.988	0.913 – 1.069	0.767	0.879
*Men*										
Adjusted by birth cohort only	568 (172)	0.986	0.893 – 1.089	0.783	–	568 (222)	0.982	0.901 – 1.071	0.680	–
Multivariable-adjusted^a^	533 (159)	0.987	0.890 – 1.096	0.811	0.879	533 (204)	0.983	0.898 – 1.076	0.709	0.879
*p for interaction sex x MDS*		0.960					0.931			
**Dementia incidence**										
*Women*										
Adjusted by birth cohort only	730 (31)	0.983	0.787 – 1.227	0.878	–	730 (126)	1.003	0.898 – 1.120	0.961	–
Multivariable-adjusted^a^	696 (29)	1.042	0.819 – 1.325	0.740	0.879	696 (123)	1.016	0.908 – 1.138	0.778	0.879
*Men*										
Adjusted by birth cohort only	568 (19)	0.887	0.660 – 1.190	0.423	–	568 (73)	0.830	0.713 – 0.967	0.017	–
Multivariable-adjusted^a^	533 (17)	0.826	0.603 – 1.130	0.232	0.826	533 (70)	0.825	0.708 – 0.963	0.014	0.231
*p for interaction sex x MDS*		0.426					0.048			

a Also adjusted for BMI, smoking history (never smoker or stopped < 10 years ago), marital status, physical activity, education.

All analyses with cancer morbidity (first event) were non-significant with HRs equal or almost equal to 1, indicating a total absence of association between the MDS and the outcome (e.g., women 20 year follow-up: HR = 0.988, 95% CI 0.913–1.069; men: HR = 0.983, 95% CI 0.898–1.076).

Regarding dementia, no association was observed in women at either 10 or 20 years (10 years: HR = 1.042, 95% CI 0.819–1.325; 20 years: HR = 1.016, 95% CI 0.908–1.138). In men, however, an inverse association appeared only at 20 years, and this finding remained robust after adjustment for all confounders (HR = 0.825, 95% CI: 0.708−0.963). With 70 dementia events in men, the study had 80% power to detect a hazard ratio of 0.80 or lower (equivalently, 1.25 or higher) per one-point increment in the MDS; the observed hazard ratio (0.83, 95% CI 0.71–0.96) lies close to this detection threshold.

No p-values for the above analyses remained significant after adjusting for multiple testing. The hazard ratios for all outcomes, by sex and follow-up period, are also displayed as a forest plot (Fig. 1).

**Fig. 1:**
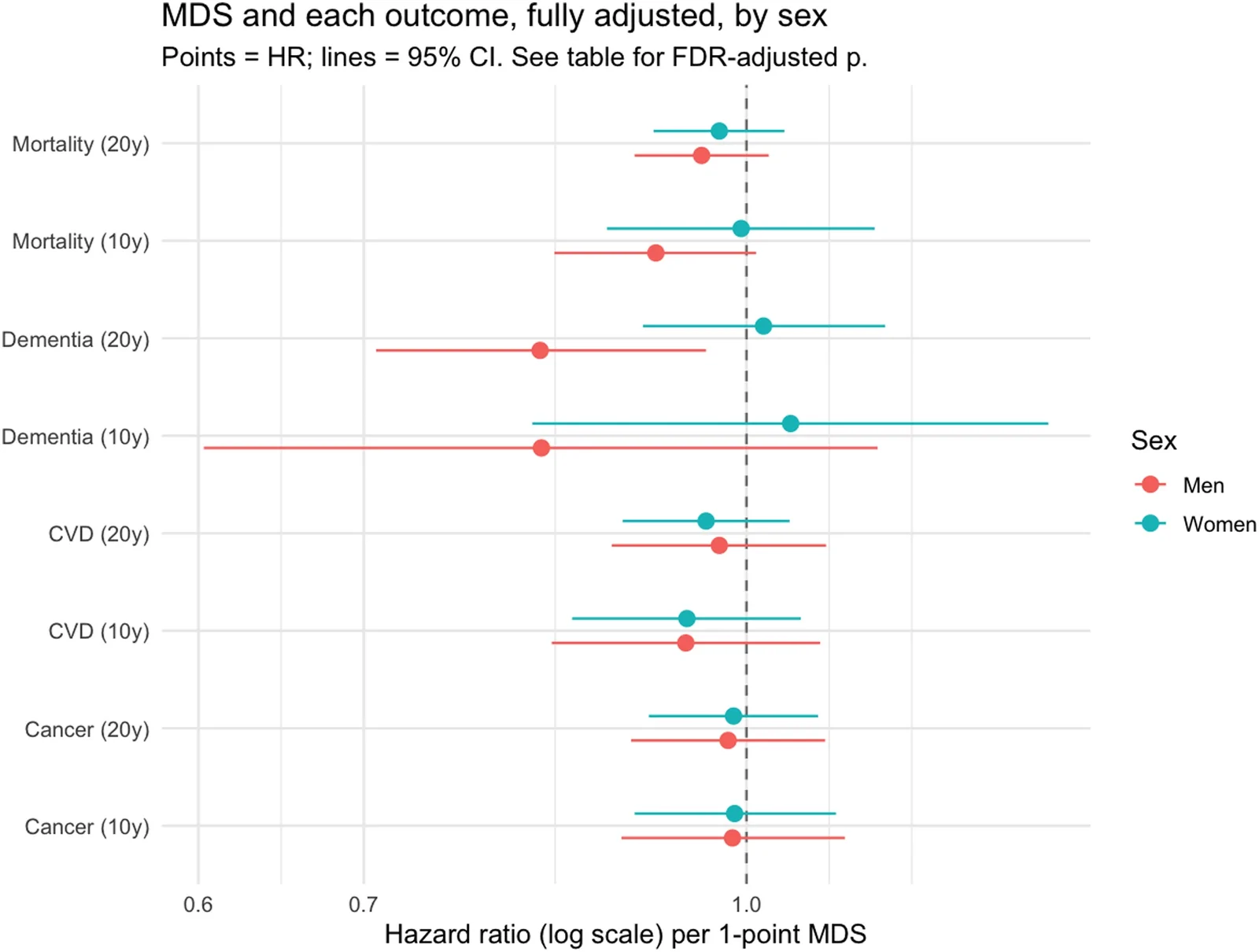
Hazard ratios for the association between the Mediterranean Diet Score (MDS) and each outcome, from fully adjusted Cox models, by sex. Each point is the hazard ratio per one-point increment in the MDS, with horizontal lines showing 95% confidence intervals, for all-cause mortality, cardiovascular disease, cancer and dementia at 10 and 20 years of follow-up, in women and men (distinguished by colour). The vertical dashed line marks a hazard ratio of 1 (no association) and the x-axis is on a logarithmic scale. Models were adjusted for birth cohort, body mass index, smoking, physical activity, marital status and education.

Cumulative dementia incidence largely overlapped in women (Fig. 2A), whereas it differed by MDS tertile in men, the highest tertile showing a lower incidence than the lower tertiles (Fig. 2B).

**Fig. 2:**
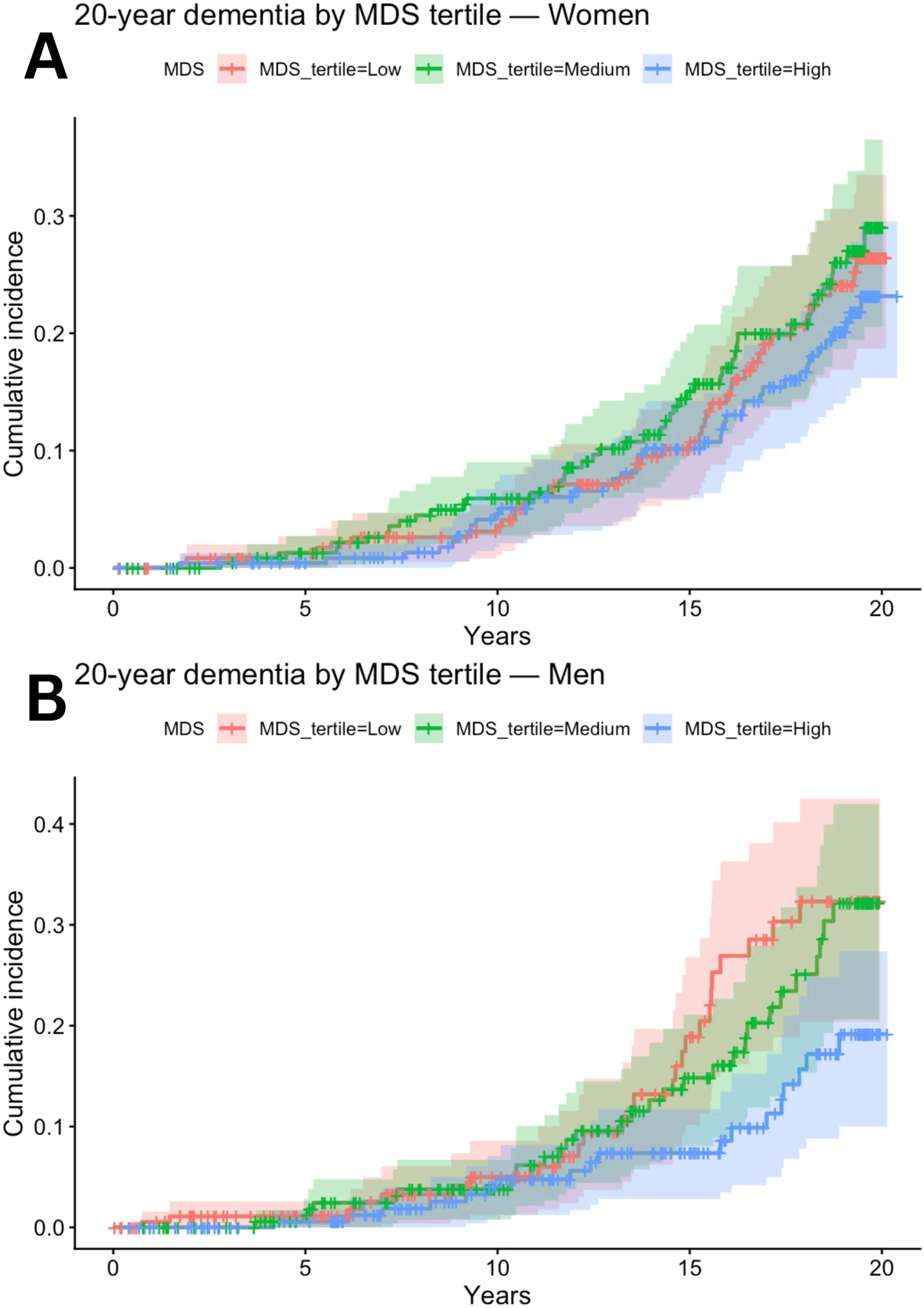
Cumulative incidence of dementia over 20 years of follow-up according to Mediterranean Diet Score (MDS) tertile, shown separately for women (A) and men (B). Curves represent the cumulative incidence of register-based dementia (one minus the Kaplan-Meier survival estimate) among participants in the low, medium and high MDS tertiles; shaded areas are 95% confidence intervals. Estimates are unadjusted. Because the MDS is a discrete score, tertile boundaries are approximate. The MDS was calculated without alcohol.

### 3.4 Proportionality of hazards

The proportional hazards assumption was assessed for all primary Cox models using Schoenfeld residuals, examining the MDS term separately from the global test. For the association between higher MDS adherence and 20-year dementia risk in men, the assumption was met for both the MDS term (p = 0.53) and the global test (p = 0.80). Where the global test suggested a departure from proportionality, most clearly for cardiovascular morbidity (global p < 0.001 at 20 years in both sexes), the MDS term nonetheless met the assumption (p ≥ 0.43), indicating that these departures reflected time-varying effects of covariates rather than of the MDS. The MDS term itself departed from proportionality in only two associations, both statistically non-significant (all-cause mortality in men at 20 years and dementia in women at 10 years, both p ≈ 0.04), which have to be interpreted with caution. Significant global tests, observed mainly for cardiovascular morbidity, were attributable to time-varying effects of covariates rather than of the exposure (MDS term p > 0.6 in all such models) and therefore did not affect inferences about diet–outcome associations.

### 3.5 Sensitivity analyses

Among participants with available information about the APOE genotype data (n = 600: 387 women, 213 men). Genotyping was not performed in the two oldest birth cohorts and was incomplete in the 1922 cohort, 26 dementia cases occurred among men and 63 among women. The association between APOE-ε4 and dementia risk showed a distinct sex-specific pattern. Among men, APOE-ε4 was a strong and significant determinant of dementia (OR 3.576; 95% CI 1.544–8.280, p = 0.003). Among women, the association was considerably weaker and did not reach statistical significance in either the 1922 (OR 1.500, 95% CI 0.481−4.677, p = 0.48) or the 1930 (OR 1.519, 95% CI 0.788–2.93, p = 0.21) birth cohorts. In men, specifically, the association between APOE-ε4 carriage and dementia appeared to differ by MDS level, with the MDS grouped as the lower two tertiles (low-to-medium) versus the upper tertile (high). Among men with low-to-medium MDS, ε4 carriers (n = 32, 10 dementia cases) had higher odds of dementia than non-carriers (n = 88, 7 cases; OR 5.26; 95% CI 1.80–15.41, p = 0.003), whereas among men with high MDS the association was attenuated and non-significant (ε4 carriers n = 28, 4 cases; non-carriers n = 65, 5 cases; OR 2.00; 95% CI 0.49–8.09, p = 0.33). The APOE-ε4 by MDS interaction was not statistically significant (p = 0.28) (Supplemeary Table 3a).

In an exploratory analysis of this subsample, men carrying the APOE-ε4 allele (i.e., the higher-risk genotype for dementia) showed a borderline significant higher mean adherence to the Mediterranean Diet than non-carriers (4.72 vs. 4.17; p = 0.06), whereas no corresponding difference was observed in women (4.52 vs. 4.44; p = 0.69). This comparison was restricted to the subsample with available APOE genotyping (n = 600 of N = 1298) and, given the resulting reduced sample size, particularly the small number of male ε4 carriers (n = 60), and limited statistical power, the finding should be regarded as hypothesis-generating and requires replication in adequately powered samples. These results are reported in Supplementary Table 3b.

Sex-by-MDS interaction tests revealed a nominally significant interaction for dementia at 20 years (p = 0.048), which suggests that the association between MDS and this outcome may differ between men and women. However, this interaction was not robust to correction for multiple testing and should therefore be interpreted with caution. Interactions were non-significant for all other 20-year outcomes and at 10-year follow-up (p ≥ 0.3).

The inverse association between MDS and dementia risk in men was robust to additional adjustment for hypertension- and diabetes-related covariates: the hazard ratio was virtually unchanged after this expanded adjustment compared with the corresponding base model in the same subsample (HR 0.839, 95% CI 0.716–0.983, p = 0.030) (Supplementary Table 4). In women, the association remained null in both the base and expanded models (p = 0.758). Supplementary analyses using false discovery rate to account for multiple testing yielded non-significant associations.

The analyses about the association between diet and the risk of dementia at the 20-year follow-up in men were confirmed using a score calculated including alcohol intakes and excluding the oldest birth cohort because of a major difference in alcohol-related survey questions compared to subsequent recruitments (Model adjusted by birth cohort only: HR = 0.845; 95% CI 0.735−0.971; Multivariable-adjusted model: HR = 0.838; 95% CI 0.727−0.965).

The association between higher MDS adherence and 20-year dementia in men was robust across several sensitivity analyses. Allowing the MDS coefficient to vary over time did not indicate any change in the association across follow-up (interaction between the MDS and follow-up time, p = 0.93), consistent with a stable effect. After multiple imputation of all missing covariate data, the estimate in men was essentially unchanged (pooled HR 0.83, 95% CI 0.71 to 0.97; 568 men, 73 events), and it remained null in women (HR 1.02, 95% CI 0.91–1.15). Estimates for all outcomes were similar to those from the complete-case analyses, and none remained statistically significant after FDR correction (Supplementary Table 5). In an analysis excluding dementia cases within the first five years of follow-up, the association in men persisted (HR 0.82, 95% CI 0.70 to 0.96; 66 events) and remained null in women (HR 1.03, 95% CI 0.92–1.16).

Finally, retaining the participants excluded for implausible energy reporting (n = 1369) left the estimates essentially unchanged for all outcomes, and the inverse association with 20-year dementia in men remained significant (HR 0.86, 95% CI 0.74 to 0.998).

## 4 Discussion

### 4.1 Discussion of methods and results

This study investigated the association between adherence to the MDS and four long-term outcomes across different elderly birth cohorts. An inverse association with dementia was observed in men only, whereas the association with all-cause mortality in men at 10 years did not persist after full adjustment. These results should be interpreted with caution, as none remained statistically significant in sensitivity analyses after correction for multiple testing.

The MDS was selected as an indicator of healthy diet patterning to maintain continuity with our earlier analysis of this cohort [22] and comparability with the wider literature. Although the Nordic and Mediterranean dietary patterns overlap substantially in their emphasis on plant foods, fish, and unsaturated fats, they differ in characteristic components. More specifically, the Mediterranean pattern is defined by olive oil, legumes, and moderate wine consumption, whereas the Nordic pattern emphasises rapeseed oil, root vegetables, berries, and wholegrain rye [33]. Also, the MDS is the more extensively validated index for cognitive and neurodegenerative outcomes.

The Mediterranean diet is a widely recognized eating pattern associated with better brain health [34], likely attributed to its multifactorial biological pathways, including the reduction of oxidative stress, the lowering of chronic inflammation, and improvements in vascular health [35]. A high adherence is robustly associated with a slower rate of cognitive decline and a lower risk of dementia [12]. However, previous findings are not entirely consistent and, importantly, diverge by sex. In the EPIC-Spain Dementia Cohort (mean follow-up 21.6 years), higher adherence was not significantly associated with overall dementia in the highest versus lowest category (HR approximately 0.80), but a significant inverse linear trend was present in the whole cohort and, on stratification, in women (per 2-point increment, HR 0.90; p for trend = 0.04), but not in men (p for trend = 0.17). This association was confined to non-Alzheimer's dementia, where it was strong in women (HR 0.74; p for trend = 0.001). Our inverse association in men, but not women, thus contrasts with this female-specific result [36]. Methodological differences may contribute: EPIC-Spain enrolled younger participants and used active clinical ascertainment that allowed dementia to be sub-typed, whereas our cohort began at age 70, relied on register-based ascertainment that misses more than half of cases and cannot reliably distinguish subtypes, and used a different diet assessment and scoring approach. Given the small number of cases, we could not examine subtypes and cannot determine whether the association in men reflects a specific subtype or mechanism. However, in Swedish cohorts results for dementia have been mixed, with a long-term study showing little or no association in the very olds [37]. Sex differences also extend to dietary habits, as Swedish women report higher intakes of vegetables, fruit, fish and wholegrain foods (core components of the MDS) and better overall diet quality than men [38]. Furthermore, the Mediterranean diet is associated with favourable changes in brain functional connectivity and may influence the risk of Alzheimer disease by interfering with amyloid-beta (Aβ) and tau pathology [39].

Several explanations for the absence of an association in women can be proposed, although none was tested directly in this study. One possibility is a lower contrast in baseline risk. Compared with men, women had a markedly lower smoking prevalence and lower all-cause mortality, reflecting their greater longevity; this more favourable baseline risk profile, together with the healthy-survivor selection that is stronger in longer-living women, may compress the range of risk across which diet can act and limit the ability to detect an association. At the same time, greater longevity means that women remained at risk of dementia for longer, which may have contributed to their higher cumulative incidence despite comparable diet adherence, while diet measured at age 70 becomes a weaker discriminator of dementia occurring decades later. These explanations should be regarded as hypotheses, and the null finding in women should not be interpreted as evidence that diet is unrelated to dementia risk in women.

In the present study, while a significant initial association between MedDiet adherence and lower all-cause mortality was observed in unadjusted models in men at 10 year follow-up, this association was not significant after comprehensive adjustment for potential confounding factors. This finding contrasts with robust evidence from a recent meta-analysis by Soltani and colleagues indicating that higher adherence is consistently associated with a reduced risk of long-term all-cause mortality in subjects recruited from different age groups, often persisting even after adjusting for potential confounders [40].

The Mediterranean diet is widely and consistently associated with a significant reduction in overall mortality, CVD incidence, and CVD mortality [41] in different age groups. The lack of significant associations with either CVD or cancer contrasts with previous trials like PREDIMED [42]. That trial, however, was a primary prevention intervention in middle-aged and older adults selected for elevated cardiovascular risk, whereas the present study is observational, begins at age 70, and is conducted in a cohort with comparatively low baseline risk and a substantial degree of prior survivorship. Because participants had already survived to age 70 without these outcomes, the cohort is depleted of the most susceptible individuals, and competing mortality further attenuates associations. Similarly, the lack of a significant link between MedDiet adherence and cancer incidence conflicts with evidence associating high adherence with a reduced risk of overall cancer incidence [43]. The Mediterranean diet is linked to prevention and management of various cancers (e.g., breast, colorectal, gastric, prostate) [[44], [45], [46], [47]]. Discrepancies often stem from differences in study design (cohort vs. case-control), populations, specific cancer types examined, and inconsistent definitions of adherence. A single dietary assessment at age 70 may be particularly limited for cancer, whose long latency reflects cumulative exposures over preceding decades rather than diet in later life.

Further research is needed to determine if the null findings observed here are due to residual confounding, methodological limitations (such as reliance on single baseline dietary assessment), or if the specific cohort lacks the necessary statistical power for incident cancer and CVD outcomes.

### 4.2 Strengths and limitations

This study has several strengths, including the use of validated diet history interviews conducted face to face with a dietician. Additional strengths are the adjustment for multiple confounders and the availability of anthropometric measures at both baseline and follow-up. Limitations include the absence of repeated dietary assessments, which likely introduces non-differential misclassification, biasing associations toward the null, and the relatively small sample size, particularly for the analyses including APOE. Also, education data were missing for 23.6% of participants in the 1922 cohort, for whom it was collected retrospectively by phone rather than at baseline. Therefore, there is a potential risk for residual confounding from unmeasured socioeconomic factors. These participants were excluded from fully adjusted models, and if missingness was related to educational level or health status, a degree of selection bias cannot be excluded. Although analyses were adjusted for birth cohort and other confounders, residual cohort effects cannot be ruled out considering that birth cohorts spanning 1901–1930 differ substantially in mortality, disease incidence and lifestyle. Moreover, selective survival bias cannot be excluded, as high-risk APOE-ε4 carriers may have died before age 70, potentially underestimating the associations. This APOE analysis was exploratory and based on a much smaller subsample (n = 600, including only 60 male ε4 carriers), since genotyping was unavailable in the two oldest cohorts; the finding in men should therefore be regarded as hypothesis-generating and requires replication in larger, adequately genotyped samples.

Another limitation is that cancer data came from a different register and only data updated to 18 year follow-up was available. Also, dementia is heavily underrepresented in register studies, which miss more than 50% of cases, particularly among home dwellers [48]. If under-ascertainment were unrelated to diet and false-positive diagnoses were rare, it would mainly reduce statistical power while leaving relative estimates approximately unbiased. Differential misclassification is, however, possible. If lower MDS were associated with a higher likelihood of moving into residential care, where cognitive decline is more likely to be recorded in registers, cases would be preferentially detected among participants with a lower MDS, which would exaggerate an inverse association. Because older men more often live with a partner than older women, and may remain at home longer despite cognitive decline, such a mechanism could also contribute to the sex difference in the observed associations. We could not examine this directly, and the dementia findings should be interpreted with this possibility in mind.

With 70 dementia events among men, the smallest hazard ratio detectable with 80% power was approximately 0.80. The observed estimate (HR 0.83) lies close to this threshold. The finding is therefore compatible with a true modest association, with a chance finding, or with insufficient precision, and our data cannot distinguish between these possibilities.

The generalisability of these findings may be limited to older adults living in high‑income countries with dietary patterns and healthcare systems similar to those of Sweden. As this study was conducted in a population‑based cohort of 70‑year‑old Swedes, caution is warranted when extrapolating the results to younger populations, non‑European settings, or contexts with substantially different dietary habits and sociodemographic characteristics.

Moreover, dietary habits between the ages of 70 and 90 are likely to change for reasons that cannot be fully captured in a cohort study, chiefly the onset of disability and disease, which may in turn reduce appetite and lead to moves into care. A relevant aspect is that information on loss of appetite, common at this age, was not available in our study and could not be included as a covariate in the analyses. Finally, the study was not pre-registered and no formal statistical analysis plan was deposited in advance. Analyses of dementia were motivated by a priori interest based on the existing literature.

### 4.3 Conclusions

In men, a higher MDS was associated with a lower risk of dementia at 20 year follow-up. This association persisted after adjustment for potential confounders and in sensitivity analyses, but did not remain statistically significant after correction for multiple comparisons. An additional inverse association with all-cause mortality was found in men only at 10 year follow-up, but disappeared after adjustment for potential confounders. A key strength of our study is the availability of high-quality dietary history data collected at age 70, information that is particularly valuable from a life course perspective, even though dietary habits may have changed over the subsequent 20 years. Further studies are needed to confirm these potential sex-specific associations in larger samples and to evaluate the potential moderating effects of sex, the potential confounding effects of socioeconomic factors not available in this study, as well as the influence of residual confounding and residual bias.

## Declaration of Generative AI and AI-assisted technologies in the writing process

Claude (Model Opus 5.5) was used solely for linguistic editing and text refinement. No generative AI tool was involved in data collection, statistical analyses, or the interpretation of results. The authors reviewed the content and further edit it if needed, and take full responsibility for the content of the published article.

## Funding sources

The H70 studies and the prospective women study 1930 sample was funded by the following grants: 10.13039/501100006636Swedish Research Council for Health, Working Life and Welfare (Forte) (2004-0145, 2006-0596, 2008-1111, 2010-0870, 2013-1202, 2018-00471, 2001-2646, 2003-0234, 2004-0150, 2006-0020, 2008-1229, 2012-1138, AGECAP 2013-2300, 2013-2496, Research Center EpiLife 2006-01506), the Swedish Research Council for Sustainable Development (2012-00038), 10.13039/501100004359Swedish Research Council (2015-0528), Swedish governmental funding of clinical research (ALF), ALF agreement in Western Sweden (964519, 720201, 508231, 429931, 138651, 11008), Stena Foundation, Swedish Research Council (11267, 2005-8460, 2007-7462, 2012-5041, 2015-02830, 2019-01096, 2022-00882, 2013-8717, NEAR 2017-00639, 2015-0528), Konung Gustaf V:s och Drottning Victorias Frimurarestiftelse, Hjärnfonden (FO2014-0207, FO2016-0214, FO2018-0214, FO2019-0163, FO2020-0235, FO2021-0213, FO2024-0341), 10.13039/501100008599Alzheimerfonden (AF-554461, AF-647651, AF-743701, AF-844671, AF-930868, AF-940139, AF-968441, AF-980935, AF-1012063), Eivind och Elsa K:son Sylvans stiftelse, Ann-Louise och Sven-Erik Beiglers stiftelse, The Alzheimer's Association Zenith Award (ZEN-01-3151), The Alzheimer's Association Stephanie B. Overstreet Scholars (IIRG-00-2159), The Bank of Sweden Tercentenary Foundation, Stiftelsen Söderström-Königska Sjukhemmet, Stiftelsen för Gamla Tjänarinnor, Handlanden Hjalmar Svenssons Forskningsfond.

## Data availability

The data used in this study derive from two population-based cohorts in Gothenburg, Sweden: the Gothenburg H70 Birth Cohort Studies and the Prospective Population Study of Women in Gothenburg (PSWG). Because these data contain potentially identifying information and are governed by Swedish data protection legislation and the terms of the relevant ethical approvals, they cannot be made publicly available. De-identified data may be made available to qualified researchers for legitimate scientific purposes upon reasonable request, subject to approval by the respective study steering committees and to a data transfer agreement. Access to the PSWG (women's) cohort data follows the study's data sharing policy; access to H70 data requires the agreement of the H70 study group.

## CRediT authorship contribution statement

**Gianluca Tognon:** Conceptualization, Formal analysis, Writing - original draft, Visualization. **Valter Sundh:** Formal analysis. **Elisabet Rothenberg:** Conceptualization, Project administration, Writing - review & editing. **Jessica Samuelsson:** Writing - review & editing. **Silke Kern:** Writing - review & editing. **Ingmar Skoog:** Writing - review & editing, Project administration, Resources. **Lauren Lissner:** Conceptualization, Supervision, Project administration, Writing - original draft, Writing - review & editing.

## Declaration of competing interest

The authors declare that they have no known competing financial interests or personal relationships that could have appeared to influence the work reported in this paper.

## Footnotes

Appendix A Supplementary material related to this article can be found, in the online version, at doi:https://doi.org/10.1016/j.jnha.2026.101012.
