# Supplementary material for: Sex-specific associations between Mediterranean Diet Score and long-term risk of mortality, dementia and other chronic diseases. 20-year evidence from a Swedish population study of 70-year old individuals
Source: J Nutr Health Aging. 2026 Oct 28;30(12):101012. doi: 10.1016/j.jnha.2026.101012 (PMC13742086; doi:10.1016/j.jnha.2026.101012)
Supplement: Supplementary file 1 [file mmc2.pdf]

A

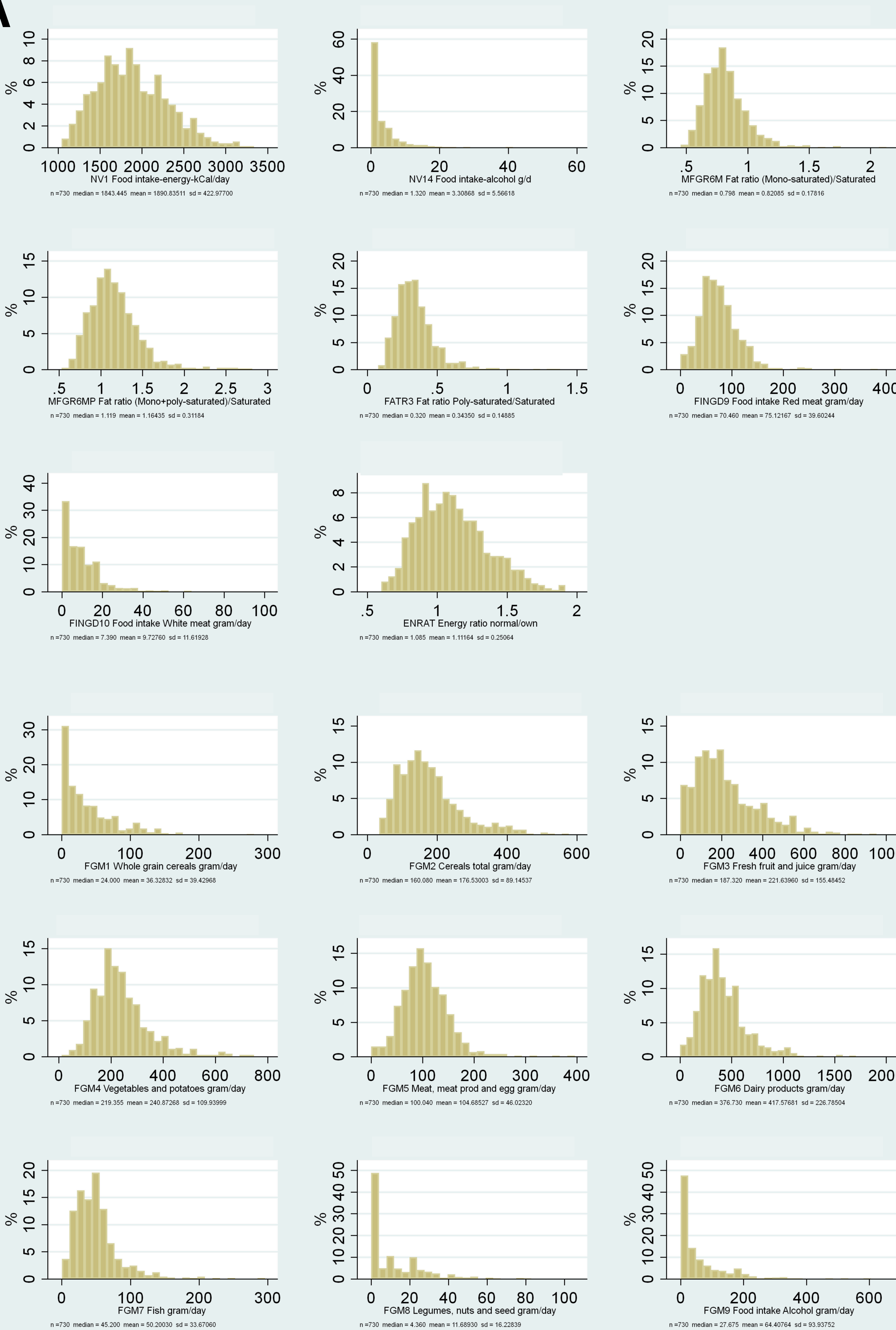
NV1 Food intake-energy-kCal/day
n =730 median = 1843.445 mean = 1890.83511 sd = 422.97700
NV14 Food intake-alcohol g/d
n =730 median = 1.320 mean = 3.30868 sd = 5.56618
MFGR6M Fat ratio (Mono-saturated)/Saturated
n =730 median = 0.798 mean = 0.82085 sd = 0.17816
MFGR6MP Fat ratio (Mono+poly-saturated)/Saturated
n =730 median = 1.119 mean = 1.16435 sd = 0.31184
FATR3 Fat ratio Poly-saturated/Saturated
n =730 median = 0.320 mean = 0.34350 sd = 0.14885
FINGD9 Food intake Red meat gram/day
n =730 median = 70.460 mean = 75.12167 sd = 39.60244
FINGD10 Food intake White meat gram/day
n =730 median = 7.390 mean = 9.72760 sd = 11.61928
ENRAT Energy ratio normal/own
n =730 median = 1.085 mean = 1.11164 sd = 0.25064
FGM1 Whole grain cereals gram/day
n =730 median = 24.000 mean = 36.32832 sd = 39.42968
FGM2 Cereals total gram/day
n =730 median = 160.080 mean = 176.53003 sd = 89.14537
FGM3 Fresh fruit and juice gram/day
n =730 median = 187.320 mean = 221.63960 sd = 155.48452
FGM4 Vegetables and potatoes gram/day
n =730 median = 219.355 mean = 240.87268 sd = 109.93999
FGM5 Meat, meat prod and egg gram/day
n =730 median = 100.040 mean = 104.68527 sd = 46.02320
FGM6 Dairy products gram/day
n =730 median = 376.730 mean = 417.57681 sd = 226.78504
FGM7 Fish gram/day
n =730 median = 45.200 mean = 50.20030 sd = 33.67060
FGM8 Legumes, nuts and seed gram/day
n =730 median = 4.360 mean = 11.68930 sd = 16.22839
FGM9 Food intake Alcohol gram/day
n =730 median = 27.675 mean = 64.40764 sd = 93.93752

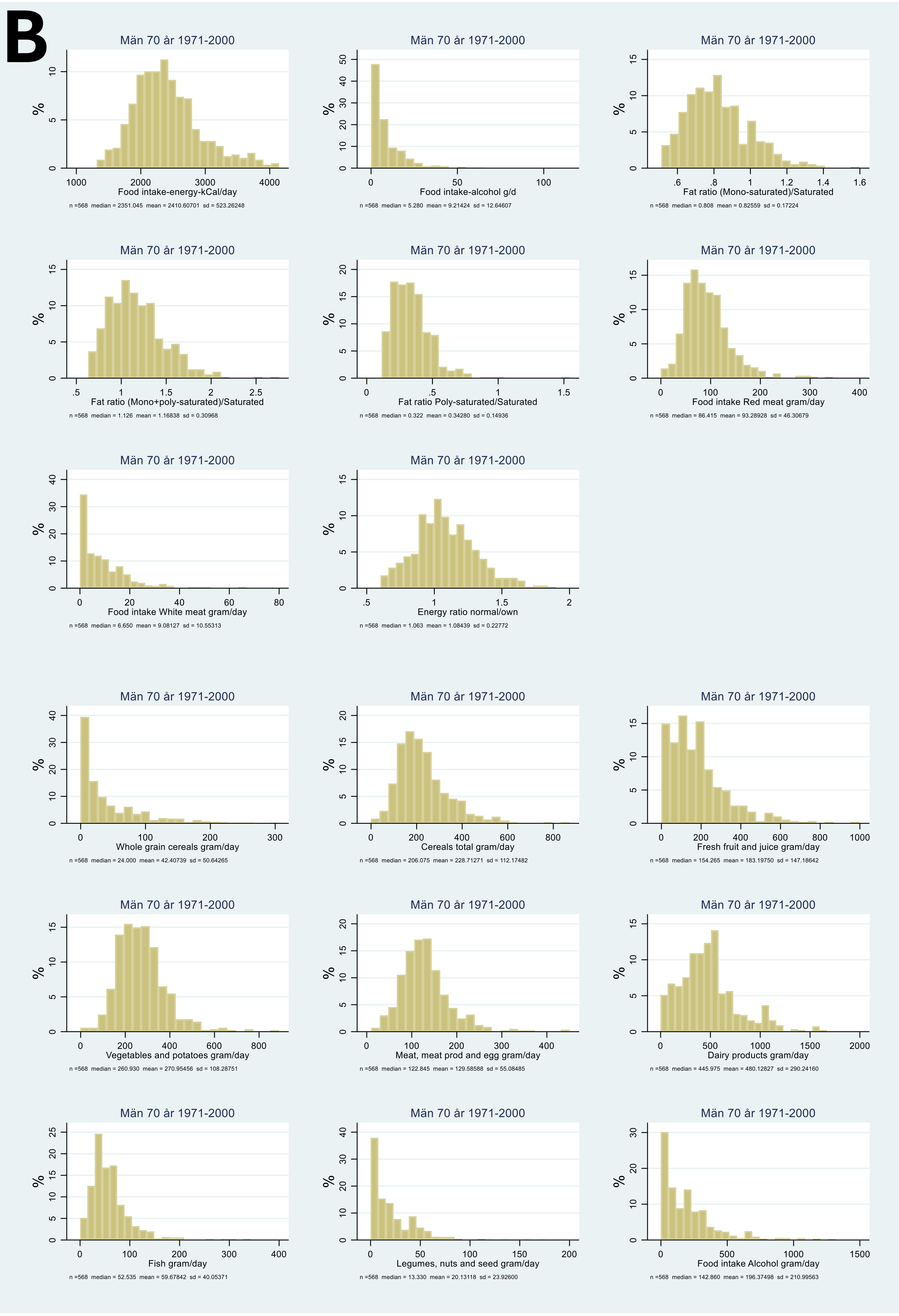
B
Män 70 år 1971-2000
Food intake-energy-kCal/day
n =568 median = 2351.045 mean = 2410.60701 sd = 523.26248
Män 70 år 1971-2000
Food intake-alcohol g/d
n =568 median = 5.280 mean = 9.21424 sd = 12.64607
Män 70 år 1971-2000
Fat ratio (Mono-saturated)/Saturated
n =568 median = 0.808 mean = 0.82559 sd = 0.17224
Män 70 år 1971-2000
Fat ratio (Mono+poly-saturated)/Saturated
n =568 median = 1.126 mean = 1.16838 sd = 0.30968
Män 70 år 1971-2000
Fat ratio Poly-saturated/Saturated
n =568 median = 0.322 mean = 0.34280 sd = 0.14936
Män 70 år 1971-2000
Food intake Red meat gram/day
n =568 median = 86.415 mean = 93.28928 sd = 46.30679
Män 70 år 1971-2000
Food intake White meat gram/day
n =568 median = 6.650 mean = 9.08127 sd = 10.55313
Män 70 år 1971-2000
Energy ratio normal/own
n =568 median = 1.063 mean = 1.08439 sd = 0.22772
Män 70 år 1971-2000
Whole grain cereals gram/day
n =568 median = 24.000 mean = 42.40739 sd = 50.64265
Män 70 år 1971-2000
Cereals total gram/day
n =568 median = 206.075 mean = 228.71271 sd = 112.17482
Män 70 år 1971-2000
Fresh fruit and juice gram/day
n =568 median = 154.265 mean = 183.19750 sd = 147.18642
Män 70 år 1971-2000
Vegetables and potatoes gram/day
n =568 median = 260.930 mean = 270.95456 sd = 108.28751
Män 70 år 1971-2000
Meat, meat prod and egg gram/day
n =568 median = 122.845 mean = 129.58588 sd = 55.08485
Män 70 år 1971-2000
Dairy products gram/day
n =568 median = 445.975 mean = 480.12827 sd = 290.24160
Män 70 år 1971-2000
Fish gram/day
n =568 median = 52.535 mean = 59.67842 sd = 40.05371
Män 70 år 1971-2000
Legumes, nuts and seed gram/day
n =568 median = 13.330 mean = 20.13118 sd = 23.92600
Män 70 år 1971-2000
Food intake Alcohol gram/day
n =568 median = 142.860 mean = 196.37498 sd = 210.99563
%
