# Supplementary material for: Sex-specific associations between Mediterranean Diet Score and long-term risk of mortality, dementia and other chronic diseases. 20-year evidence from a Swedish population study of 70-year old individuals
Source: J Nutr Health Aging. 2026 Oct 28;30(12):101012. doi: 10.1016/j.jnha.2026.101012 (PMC13742086; doi:10.1016/j.jnha.2026.101012)
Supplement: Supplementary file 2 [file mmc1.docx]

**SUPPLEMENTARY TABLES**

**Supplementary Table 1:** Comparison of the baseline characteristics of participants included in the analyses (n = 1,298) with those excluded based on energy intake-to-basal metabolic rate (EI/BMR) ratio.

|  | **n (%)** | | **p-value** |
| --- | --- | --- | --- |
|  | **Included (n=1,298)** | **Excluded (n=71)** |  |
| Sex |  |  | 0.25 |
| *Women* | 730  (56.2 %) | 35  (54.0%) |  |
| *Men* | 568  (43.8%) | 36  (50.7%) |  |
| Birth cohort |  |  | 0.78 |
| *Born 1901-02* | 348  (26.8%) | 21  (26.9%) |  |
| *Born 1911-12* | 248  (19.1%) | 12  (16.9%) |  |
| *Born 1922* | 178  (13.7%) | 12  (16.9%) |  |
| *Born 1930* | 524  (40.4%) | 26  (36.6%) |  |
| Education above basics | 378  (29.1%) | 20  (28.2%) | 0.86 |
| Married | 800  (61.6%) | 41  (57.7%) | 0.53 |
| Current/recent smoker | 394  (30.4%) | 23  (32.4%) | 0.69 |
| Low physical activity | 180  (13.9%) | 18  (25.4%) | 0.014 |
|  | **Mean (SD)** | |  |
| BMI (kg/m^2^) | 26.5  (4.0) | 26.4  (5.6) | 0.85 |
| MDS without alcohol (main) | 4.02  (1.59) | 3.78  (1.50) | 0.20 |
| MDS with alcohol | 4.53  (1.73) | 4.17  (1.61) | 0.09 |

**Supplementary Table 2:** Summary of the food items included in each food groups that were used for the calculations of the Mediterranean Diet Score.

| **Group Name** | **High-Level Categories (Intermediate Grouping)** | **Detailed Food Items Included (Examples)** |
| --- | --- | --- |
| Whole Grain Cereals | Hard bread and Whole grain bread. | Includes Crispbread (Knäckebröd, Finn Crisp, Husman Knäcke Bröd Hårt, Special gluten-free crispbread), Hard Breads (various Rye and Wheat hard breads, often specified by fiber percentage, such as Fiber Bröd Hårt), and Unsweetened Whole Grain Bread (Fullkornbröd Osötat). |
| All Grain Cereals | Hard bread, Soft coarse bread, Whole grain bread, White bread, Swedish rye bread (Kavring), Rusks, Porridge (cooked with water), Gruel, Muesli, Rice, Spaghetti/Macaroni, Wheat flour, Bran, and Cornflakes. | Breads/Rusks: All types of hard bread, soft coarse breads (Råg/Vete bröd), White bread, hamburger/sausage buns, various rusks (Veteskorpor, Rågskorpor), and soft flatbread (Tunnbröd). Cereals/Porridge/Gruel: Cornflakes (berikade/med sockerlager), Muesli, various porridges (Oatmeal/Havregrynsgröt, Buckwheat porridge/Bovete gröt, Graham porridge), and Gruel for adults and children (Välling). Grains/Pasta: Wheat flour, Bran (Vetekli, Havrekli), various rice types (polished, brown, sticky, cooked), and Pasta (macaroni, spaghetti, egg noodles, couscous). |
| Fresh Fruit and Juice | Fresh fruit (other), Orange/Citrus, Banana, Apple and pear, and Juice. | Fruit: Apples (with or without peel), Pears, Bananas, Citrus fruits (Oranges, Lemons, Clementines, Grapefruit), Avocado, Melon (Honeymelon, Watermelon), Pineapple, Apricots, Berries (Strawberries/Jordgubbar, Raspberries/Hallon, Lingon, Blackcurrants), Kiwi, Plums, Peaches, Nectarines, Grapes (Vindruvor), and Sharon/Kaki fruit. Juice/Nectar: Includes orange juice, apple juice, grape juice, vegetable juice (Grönsaksjuice), and various fruit nectars. |
| Vegetables and Potatoes | Vinegar pickle olives, Fresh vegetables, Cooked vegetables and vegetable dishes, Salad, Soups, Potato dishes, Boiled potato, Fried potato, and Deep fried potato. | Vegetables (Fresh/Cooked): Tomatoes, Cucumbers, Carrots, Onions, Leeks, various lettuces (Iceberg, Head lettuce, Chinese cabbage), Broccoli, Cauliflower, Brussels sprouts, Spinach, Zucchini, Mushrooms (Champinjoner, Kantareller), Peas, Green beans, Asparagus, and root vegetables like Swede (Kålrot) and Parsnip (Palsternacka). Prepared Dishes: Soups (Vegetable soup, Tomato soup, Creamy/Clear soups), Vegetable stews and casseroles (Moussaka Vegetarisk, Grönsaksgratäng), and prepared vegetable mixes (e.g., American blend, Summer blend). Potatoes & Pickled: Boiled potato (with or without peel), Fried potato, Mashed potato, Potato gratin, Croquettes, and Deep-fried potatoes (Pommes Frites). Also includes pickled items like Olives (green/black), Pickled cucumber (Ättiksgurka, Saltgurka), and Pickled beetroot (Rödbeta inlagd). |
| Meat, Meat Products and Egg | Cold cuts (meat, sausage, liver paste), Minced meat, Pork, Beef/Lamb etc., Chicken/Poultry, Liver/kidney, Egg, and Black pudding. | Meats: Beef (Nöt, Ox), Pork (Griskött, Bacon, Skinka/Ham, Fläsk), Lamb (Lamm), Veal (Kalv), Poultry (Chicken/Kyckling, Turkey/Kalkon), and Game (Elk/Älg, Reindeer/Ren) in various forms (raw, fried, grilled, boiled). Products: Minced meat (Köttfärs), Meatballs (Köttbullar), Meatloaf (Köttfärslimpa), Black pudding (Blodpudding), Liver paste (Leverpastej), Cold cuts (Salami, Medvurst, Påläggskorv), and Bacon (steamed or fried). Offal: Liver and Kidney (Kalv/Nöt/Kyckling lever). Egg: Raw, boiled, fried, and included in dishes like Omelettes, Scrambled egg (Äggröra), and Egg standing (Äggstanning). |
| Dairy products | Cheese, Whey cheese/butter, Cream (<20% fat), Yoghurt/Sour milk/Milk (3% fat), Light milk and light sour milk, Cream (>20% fat), Fruit yoghurt, Medium fat milk, Minimal fat milk, and Cottage cheese/Quark. | Milk & Soured Products: Milk (various fat contents: H-mjölk 3%, Mellanmjölk 1.5%, Lättmjölk 0.5%, Minimjölk <0.1%), Fil (Sour milk), A-Fil, and Dofilus. Yoghurt: Natural yoghurt, Fruit yoghurt, and Light fruit yoghurt. Cream & Cream Products: Whipped cream (Vispgrädde), Coffee cream (Kaffegrädde), Cooking cream (Matlagningsgrädde), and Crème Fraiche (including light versions). Cheese & Paste: Hard cheese (Ost) with fat content ranging from 10% to 31%, Soft cheeses (Brie, Fetaost, Camembert), Processed cheese (Smältost), Cottage cheese (Keso), Quark (Kesella), and Whey cheese/paste (Mesost/Messmör). |
| Fish | Caviar, Shellfish, Fish spread/cold cut, Boiled lean fish, Fried lean fish, Herring, Mackerel, Salmon and other fatty fish, and other fish. | Fish: Fatty fish (Salmon/Lax, Mackerel/Makrill, Herring/Sill, Eel/Ål) and lean fish (Cod/Torsk, Haddock/Kolja, Plaice/Rödspätta) prepared in various ways (boiled, fried, smoked, pickled). Shellfish: Shrimp/Prawns (Räkor), Mussels (Musslor), Crab (Krabba), Lobster (Hummer), and Crayfish (Kräfta). Products: Fish balls (Fiskbullar), Fish spread/cold cuts, Fish gratins, Fish soup, Tuna (Tonfisk), Sardines, Anchovies (Ansjovis), and Roe/Caviar (Kaviar, Löjrom, Torskrom). |
| Legumes, Nuts and Seed | Legumes. | Legumes: Beans (Brown, Green, Red, White, Soybeans), Peas (Green, Yellow/ärtor), Chickpeas (Kikärter), and Lentils (Linser). This group also includes dishes based on legumes, such as Pea soup (Ärtsop pa) and Lentil soup (Linssop pa). Nuts and Seeds: Cashew nuts, Hazelnuts, Peanuts, Walnuts, Pistachios, Almonds (Sötmandel), Sunflower seeds, Flax seeds (Linfrön), and Sesame seeds. |
| Alcohol | Light beer, Light wine, Medium-strength beer, Strong beer, Fortified wine, and Spirits. | Beer: Weak drinks (Svagdricka), Light beer (Lättöl), Pilsner, Medium-strength beer (Folköl), and Strong beer (Starköl). Wine: Red wine (Rödvin), White wine (Vitt Vin), Rosé wine, and Light wine (Lättvin). Spirits/Fortified: Fortified wine (Starkvin), Madeira, Sherry, Port wine, Fortified wine glögg (Starkvinsglögg), Spirits (Starksprit), Brandy/Cognac (Konjak), Vodka (Brännvin), Whisky, Gin, Rum, and Liqueur (Likör). |

**Supplementary Table 3a:** Mediterranean Diet Score (calculated without considering alcohol intakes) by APOE ε4 carrier status, stratified by sex (exploratory subsample analysis).

| **Sex** | **APOE ε4 status** | **N** | **Mean MDS (SD)** | **p-value** |
| --- | --- | --- | --- | --- |
| **Women** | Non-carrier | 275 | 4.44 (1.63) | 0.693 |
|  | Carrier | 112 | 4.52 (1.58) |  |
| **Men** | Non-carrier | 153 | 4.17 (1.56) | 0.060 |
|  | Carrier | 60 | 4.72 (1.68) |  |

**Supplementary Table 3b:** Mediterranean Diet Score (calculated without considering alcohol intakes) by APOE ε4 carrier status, stratified by MDS levels.

| **MDS group** | **APOE-ε4 status** | **n** | **Dementia cases (%)** | **OR**  **(95% CI)** | **p** |
| --- | --- | --- | --- | --- | --- |
| **Low-to-medium** | Non-carrier | 88 | 7 (8.0) | 1 (ref) |  |
|  | Carrier | 32 | 10 (31.3) | 5.26 (1.80–15.41) | 0.003 |
| **High** | Non-carrier | 65 | 5 (7.7) | 1 (ref) |  |
|  | Carrier | 28 | 4 (14.3) | 2.00 (0.49–8.09) | 0.33 |

**Supplementary Table 4:** Sensitivity analysis of the association between Mediterranean Diet Score and dementia incidence, with additional adjustment for hypertension- and diabetes-related covariates, by sex. ^+^also including hypertension & diabetes markers

| **Sex** | **Model** | **N (dementia events)** | **HR for MDS (95% CI)** | **p-value** |
| --- | --- | --- | --- | --- |
| **Men** | Adjusted by birth-cohort only | 568 (73) | 0.843  (0.724–0.981) | 0.028 |
|  | Multivariate-adjusted model^+^ | 553 (69) | 0.839  (0.716–0.983) | 0.030 |
| **Women** | Adjusted by birth cohort only | 730 (126) | 1.018  (0.910–1.137) | 0.758 |
|  | Multivariate-adjusted model^+^ | 715 (122) | 0.997  (0.890–1.117) | 0.954 |

**Supplementary Table 5:** Sensitivity analysis of the association between the Mediterranean Diet Score (MDS) and the risk of all-cause mortality, CVD, cancer, and dementia at 10 and 20 years of follow-up, by sex: complete-case analysis compared with multiple imputation of all missing covariate data. Hazard ratios per one-point increment in the MDS, adjusted for year of birth, BMI, smoking, physical activity, marital status, and education. Multiple imputation was performed separately by sex and outcome using predictive mean matching (20 imputations), with an imputation model including all covariates, the MDS, the event indicator and the Nelson-Aalen estimate of the cumulative hazard; estimates were combined using Rubin’s rules. p-values are not adjusted for multiple testing; no association remained statistically significant after false discovery rate correction.

| **Outcome** | **Follow-up (years)** | **Sex** | **Complete-case analysis** | | | **Multiple imputation** | | |
| --- | --- | --- | --- | --- | --- | --- | --- | --- |
|  |  |  | **N (events)** | **HR for MDS (95% CI)** | **p-value** | **N (events)** | **HR for MDS (95% CI)** | **p-value** |
| All-cause mortality | 10 | Men | 533 (202) | 0.919  (0.836–1.009) | 0.077 | 568 (212) | 0.914  (0.833–1.004) | 0.059 |
|  |  | Women | 696 (113) | 0.995  (0.878–1.127) | 0.936 | 730 (127) | 0.985  (0.875–1.109) | 0.805 |
|  | 20 | Men | 533 (440) | 0.959  (0.901–1.021) | 0.194 | 568 (467) | 0.960  (0.902–1.021) | 0.190 |
|  |  | Women | 696 (440) | 0.975  (0.917–1.036) | 0.413 | 730 (464) | 0.985  (0.928–1.046) | 0.616 |
| CVD | 10 | Men | 533 (125) | 0.945  (0.834–1.071) | 0.375 | 568 (134) | 0.937  (0.829–1.059) | 0.295 |
|  |  | Women | 696 (144) | 0.946  (0.850–1.052) | 0.304 | 730 (151) | 0.938  (0.845–1.042) | 0.232 |
|  | 20 | Men | 533 (195) | 0.975  (0.882–1.077) | 0.619 | 568 (209) | 0.970  (0.880–1.069) | 0.540 |
|  |  | Women | 696 (286) | 0.963  (0.891–1.041) | 0.344 | 730 (301) | 0.957  (0.887–1.033) | 0.260 |
| Cancer | 10 | Men | 533 (159) | 0.987  (0.890–1.096) | 0.811 | 568 (172) | 0.986  (0.891–1.091) | 0.777 |
|  |  | Women | 696 (183) | 0.989  (0.901–1.087) | 0.824 | 730 (193) | 0.987  (0.900–1.082) | 0.783 |
|  | 20 | Men | 533 (204) | 0.983  (0.898–1.076) | 0.709 | 568 (222) | 0.983  (0.900–1.073) | 0.694 |
|  |  | Women | 696 (256) | 0.988  (0.913–1.069) | 0.767 | 730 (267) | 0.990  (0.916–1.070) | 0.794 |
| Dementia | 10 | Men | 533 (17) | 0.826  (0.603–1.130) | 0.232 | 568 (19) | 0.864  (0.619–1.205) | 0.352 |
|  |  | Women | 696 (29) | 1.042  (0.819–1.325) | 0.740 | 730 (31) | 1.049  (0.820–1.341) | 0.693 |
|  | 20 | Men | 533 (70) | 0.825  (0.708–0.963) | 0.014 | 568 (73) | 0.831  (0.712–0.971) | 0.021 |
|  |  | Women | 696 (123) | 1.016  (0.908–1.138) | 0.778 | 730 (126) | 1.022  (0.913–1.145) | 0.701 |
